# Estimated contributions and future mitigation strategies for HIV risk around funeral practices in western Kenya: a mathematical modeling study

**DOI:** 10.1186/s12916-025-03907-4

**Published:** 2025-02-12

**Authors:** Samuel M. Mwalili, Duncan K. Gathungu, Josiline Chemutai, Evalyne Musyoka, Daniel Bridenbecker, Clark Kirkman, David Kaftan, Hae-Young Kim, Ingrida Platais, Anna Bershteyn

**Affiliations:** 1 Strathmore University, Ole Sangale Road, P.O. Box 59857-00200, Nairobi, Kenya; 2 Jomo Kenyatta University of Agriculture and Technology, P.O. Box 62000-00200, Nairobi, Kenya; 3 Institute for Disease Modeling at the Bill & Melinda Gates Foundation, 500 5th Avenue North, Seattle, WA USA; 4 New York University Grossman School of Medicine, 227 East 30th St., New York, NY USA

**Keywords:** HIV/AIDS, Social network, Funeral, Structural interventions, Pre-exposure prophylaxis

## Abstract

**Background:**

A *disco matanga*, or “disco funeral,” is a celebration of a decedent’s life that is culturally important in parts Africa, often involving overnight travel and alcohol consumption. These are known risk factors for HIV, which is prevalent in many areas where *disco matanga* is practiced. However, the contribution of *disco matanga* to HIV transmission is not well-understood. We used agent-based network modeling to estimate how *disco matanga* impacted HIV transmission, and to explore the impact of relevant biomedical, biobehavioral, and structural interventions to reduce HIV risk.

**Methods:**

We adapted EMOD-HIV, a previously validated network-based model of HIV in the Nyanza region of Kenya, to incorporate *disco matanga* assumptions informed by literature review. Occurrence of *disco matanga* was modeled to occur following any death in the population. We compared past HIV incidence (1980–2024) with and without incorporating *disco matanga*, and future HIV incidence (2025–2050) with different interventions for *disco matanga* attendees: (1) biomedical (HIV prophylaxis), (2) biobehavioral (reduction in condomless sex partners), (3) structural (female empowerment to avoid unwanted sex). We estimated HIV infections and deaths averted in the overall population, with sensitivity analysis around intervention uptake.

**Results:**

Over 1980–2024, *disco matanga* contributed 7.8% (95% CI: 5.5–9.3%) of all HIV infections, an effect that peaked at 9.9% (95% CI: 6.4–12.0%) in the year 2004, coinciding with a peak in all-cause mortality due to HIV/AIDS. Biomedical prevention at *disco matanga* could avert up to 9.7% (95% CI: 8.9–10.5%) of adult HIV infections and 2.3% (95% CI: 1.9–2.6%) of deaths; biobehavioral 2.9% (95% CI: 2.1–3.6%) of infections and 0.9% (95% CI: 0.6–1.2%) of deaths; and structural 1.2% (95% CI: 0.5–1.8%) of infections and 0.5% (95% CI: 0.2–0.7%) of deaths. Results were highly sensitive to intervention uptake.

**Conclusions:**

We conducted the first modeling study, to our knowledge, simulating the interactions between *disco matanga*, HIV/AIDS, and intervention options. We found that biomedical, biobehavioral, or structural interventions implemented during *disco matanga* could substantially reduce HIV transmission and mortality in the Nyanza region. Research is needed to understand the feasibility and acceptability of HIV interventions tailored to local cultural practices.

**Supplementary Information:**

The online version contains supplementary material available at 10.1186/s12916-025-03907-4.

## Study design

Disco matanga, or “disco funerals,” are celebrations of a decedent’s life that are culturally important in parts Africa and often involve overnight travel and alcohol consumption. These are known risk factors for HIV. This study explores the bi-directional interactions between “disco funerals” and the HIV/AIDS pandemic, and the potential impact of biomedical, biobehavioral, and structural interventions.

## Background

A “*disco matanga*,” or disco funeral, is a cultural event practiced in parts of sub-Saharan Africa that blends mourning for a decedent with a celebration of their life [1]. Extended family members typically travel to the family’s ancestral area of residence and partake in multiple days of dancing, communal meals, overnight vigils, and fundraising to cover funeral costs. Young women face particularly strong cultural expectations to travel to *disco matanga*in order to participate in dancing, assist with food preparation, and express mourning and respect for the deceased [1].

Despite its cultural importance, *disco matanga*may exacerbate the risk of sexually transmitted infections including HIV [1]. *Disco matanga*involves known HIV risk factors such as overnight travel [2], alcohol consumption [3], recreational drug use [4], and at times unprotected sex that is coerced, transactional, and/or involving multiple partners [5]. Examples include the use of transactional sex to settle debts or raise funds. Reports of coerced sex and gang rape in the context of *disco matanga*have led some local leaders to place limitations on the practice [5–7]. For example, in the Busia district of Uganda, a 2021 regulation banned the involvement of children in *disco matanga*and restricted activities to daytime hours [8].

HIV risk in the context of *disco matanga* is particularly significant due to geographic overlap between the practice of *disco matanga* and high rates of HIV infection. In parts of western Kenya where *disco matanga*is commonly practiced, one in four adults is living with HIV [9], making unprotected sexual encounters particularly high-risk. Moreover, over history HIV and *disco matanga* may have perpetuated one another, where HIV has increased overall mortality rates, especially in the era prior to widespread HIV treatment availability. Increased mortality leads to more *disco matanga* events, which in turn may perpetuate HIV transmission. In the modern era, HIV-related mortality has been substantially reduced by HIV treatment, yet *disco matanga* may continue to be an important environment where HIV risk is elevated, and HIV prevention interventions are needed.

A wide range of interventions are available for populations at risk of HIV exposure. Biomedical interventions, which focus on the provision of biomedical tools such as HIV chemoprophylaxis via oral pill [10], injection [11], or vaginal ring [12], reduce HIV acquisition risk. Behavioral and biobehavioral interventions involve risk reduction through behavior modification, with or without the assistance biomedical tools, and may include increasing the use of condoms during sexual encounters [13], reducing numbers of sexual partners [14], and/or reducing alcohol and drug use [15]. Finally, structural interventions modify the social, economic, and/or political environments that contribute to health risks and disparities [16], such as female empowerment to avoid unwanted sexual encounters [17]. While these have been tested in a range of African settings, none to our knowledge has been empirically tested or modeled in the context of *disco matanga* [18, 19].

This study aimed to explore the HIV risk and potential HIV interventions in the context of *disco matanga* using mathematical modeling. It focuses on six counties in western Kenya comprising the former province of Nyanza, which includes counties with the highest HIV prevalence observed in Kenya, and where *disco matanga* is widely practiced. We adapted a previously validated network-based HIV transmission model of the Nyanza by incorporating *disco matanga*, with assumptions informed by literature review and the contextual expertise of the authors. We assessed the contribution of *disco matanga* to the HIV/AIDS pandemic and the potential future impact of biomedical, biobehavioral, and structural interventions.

## Methods

### Modeling framework

Epidemiological MODeling HIV (EMOD-HIV) is an agent-based network simulation model capturing sexual and mother-to-child HIV transmission, HIV disease progression, and engagement in care and prevention. Source code [20] and model documentation [21] are available online, and the model has been documented extensively in published literature [22, 23], including detailed descriptions of transmission assumptions using a modeled social network [24, 25]. Briefly, the model incorporates four sexual relationship types (marital, informal, transitory, and commercial) with different probabilities of multiple sex partners (marital: lowest, commercial: highest), durations over which partnerships are maintained (marital: longest, commercial: shortest), condom use probability within partnerships (marital: lowest, commercial: highest), and age patterns of partnerships based on HIV surveillance and biobehavioral studies. Sexual transmission is modeled at the level of individual coital acts within relationships, with transmission probability modifiers including whether a condom was used, whether an HIV-negative partner used HIV chemoprophylaxis, whether an HIV-positive partner achieved viral load suppression with antiretroviral therapy, whether an HIV-negative male partner was circumcised, whether either partner has an active sexually transmitted infection, and whether a condom was used.

EMOD-HIV model has been calibrated to HIV epidemiological data in several African settings, including the six counties surrounding Kisumu, Kenya, which constitute the former province of Nyanza. Two rural counties in this region, Homa Bay and Siaya, have overall HIV prevalence of 25%, with some communities having even higher prevalence of 30–40% [9, 26, 27]. Sexual behavior assumptions for which data are not directly available were previously calibrated using stochastic optimization so that the overall HIV epidemiological outputs, include age/sex patterns of HIV prevalence and incidence, coincide with data from population-based surveillance [28]. A description of the calibration methods and details previous calibration of EMOD-HIV to western Kenyan HIV data, including final model parameter values, is publicly available [29, 30]. The model for Nyanza was previously validated by predicting HIV incidence in 16 communities in Nyanza enrolled in a blinded, community-randomized, controlled trial of HIV treatment as prevention [31]. For future projections through 2050, we used a baseline model projection that assumed continuation of current rates of HIV diagnosis, care engagement, voluntary made medical circumcision (VMMC), HIV prophylaxis use, and use of condoms in the general population.

### Model structure adaptation for disco matanga

**Fig. 1:**
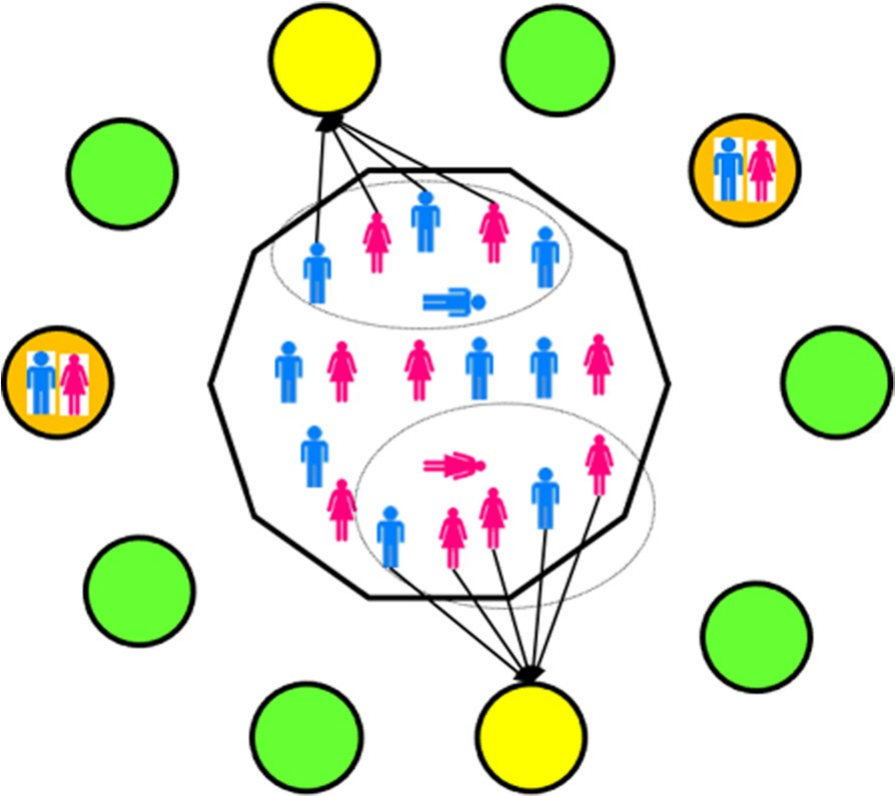
Illustration of how EMOD-HIV was adapted to incorporate *disco matanga*. The central area (“node”) contains the population that is not currently attending *disco matanga*. The model structure was modified to include disco *matanga* nodes (circles) which are vacant until used for a *disco matanga* (green circles). Deaths in the population (horizontal figures) trigger individuals to migrate to *disco matanga* “nodes” (yellow circles). Individuals actively attending *disco matanga* (orange circles) do not consummate their usual relationships but may participate in sexual contacts with other attendees. Upon conclusion of a *disco matanga*, individuals return to the central “node” and resume their usual relationships, and the node becomes vacant for re-use in a future *disco matanga* (green circles)

To model the estimated contribution of *disco matanga* to the spread of HIV/AIDS, we adapted EMOD-HIV by adding a new social network component representing sexual interactions at *disco matanga* (Fig. 1).

We leveraged a capability of the EMOD software known as “migration” in which individuals are temporarily moved to a specific social network group, known in the software as a “node” because it can be distributed across multiple computational nodes if desired. This capability of the EMOD framework has previously been leveraged in EMOD-HIV to model migration of long-distance truck drivers in Kenya [29] and has been used in other EMOD-based disease models, such as EMOD-malaria, to simulate geospatial patterns of human and mosquito migration [32].

In our implementation of *disco matanga*, individuals are “migrated” to a *disco matanga* node immediately the event of death of a different individual in the model. Thus, the proportion of the population participating in *disco matanga* was not fixed, but dependent on all-cause mortality rates at the time, multiplied by the assumed number of attendees per each *disco matanga*. Individuals attending a *disco matanga* were placed together in a social mixing group (known as a “node”) within the model. If attendees had active relationships prior to entering the *disco matanga*, and their partner(s) did not enter the same disco matanga, then pre-existing relationship(s), such as marriages, were assumed to “pause” while individuals migrate to a disco matanga, meaning that the partnerships would be “remembered” but would not be consummated for the duration of the *disco matanga* if one partner but not the other attends. Consummation patterns would resume after the conclusion of the *disco matanga*. If both partners simultaneously attended the same *disco matanga*, then consummation of the relationship would continue.

Within the *disco matanga*, new relationships were able to form and be assigned properties such as condom usage, coital act rates, and maximum number of concurrent relationships. These sexual relationships were assumed to terminate at the conclusion of the *disco matanga*, and individuals subsequently return to participating in pre-existing sexual relationships. We further implemented biomedical, biobehavioral, and structural interventions which, when active, modified condom usage, number of concurrent partners, and number of sexual acts within a *disco matanga* node. Parameters including the size and demographics of *disco matanga* participants, sexual behavior in the absence of intervention, and effects of interventions were determined using literature review, as described below.

### Model assumptions

We reviewed literature and leveraged contextual expertise from authorship team to develop model assumptions (Table 1) about *disco matanga*.

**Table 1:** Model parameter descriptions, values, and sources

Parameter	Units	Value	Source
Sexually active attendees per *disco matanga*	Number	30 (default)	[5]
Age of attendees: male	Years	12–39	[1, 5, 33]
Age of attendees: female	Years	13–49	[1, 5, 33]
Unique partners per sexually active attendee	Number	1–5 (median: 3)	Assumed
Reduction in HIV acquisition rate from biomedical intervention	Percent	95%	[34, 35]
Percent of *disco matanga* participants benefitting from biomedical intervention	Percent	30%, 70%, 100%	[36]
Reduction in unprotected coital acts from biobehavioral intervention	Percent	10%, 90%	Assumed
Reduction in sexual participation in *disco matanga*	Percent	33%	[37]

Literature suggested that *disco matangas*are attended by approximately 30 sexually active individuals, primarily adolescent and adult men and unmarried women, ranging from approximately 16 to 37 years of age, with smaller numbers of children and married or widowed women [1, 5, 33]. Girls have been observed engaging in sexual activities as early as age 12, and boys as early as age 14. A significant portion of girls, approximately 43%, reported coerced sex [1]. Reports of transactional sex were also common, e.g., male participants were reported purchasing small items such beverages and snacks for young women with the intention of instigating a sexual encounter [1, 38]. In some cases, auctions were held for the purpose of fundraising, with bids placed on a young woman. These risks are further amplified by frequent alcohol and drug use, which has been shown to be associated with risky sexual behavior including condomless sex [39–41] and multiple sexual partners [39] in western Kenya.

We further developed model assumptions regarding the potential impact of HIV prevention interventions. There is a lack of studies focused on HIV prevention for *disco matanga* specifically, but studies have examined HIV prevention in other settings where high-risk sexual encounters are likely to occur, such as bars [42] and other alcohol-serving social venues [43], border crossing [44] and other truck stops locations [45], and lodgings for itinerant workers [46]. HIV prevention interventions fell into the broad categories of (a) biomedical interventions such as antiretroviral chemoprophylaxis for HIV-negative individuals [47], (b) biobehavioral interventions, such as strategies to reduce numbers of sexual partners and increase condom use [48], and (c) social and structural interventions such as empowering women, youth, and other at-risk populations to avoid risky or unwanted sexual encounters [49].

Biomedical HIV prevention can be highly effective when taken as directed [34, 35], but effectiveness is adherence-dependent, and adherence varies widely [36]. We explored a scenario of 70% of *disco matanga* attendees using HIV prophylaxis effectively, where effective use conferred 95% reduction in HIV acquisition per coital act. We then conducted a wide sensitivity analysis of effective use ranging from 30 to 100% use of HIV prophylaxis. Biobehavioral HIV prevention is highly context-dependent and has not been tested at *disco matanga*, and the authors could come to no consensus on the plausible magnitude of biobehavioral prevention in this context. Accordingly, we conducted a bounding analysis of two extreme assumptions: 90% reduction in coital acts (high end) and 10% reduction (low end). Finally, structural interventions, such as empowerment, are not well-studied in relation to *disco matanga*, but their effects on HIV acquisition in other contexts have been moderate. For example, in a pooled analysis of 31 countries in sub-Saharan Africa, female empowerment was associated with greater ability to refuse sex (OR = 1.78, 95% CI 1.72–1.85) [37]. We explored a single assumption that empowerment may enable one-third (33%) of participants to not participate in sexual activities in the context of *disco matanga*, which can encompass a range of behaviors including non-attendance, sexual abstinence while attending, or modifications to attendance such as avoiding nighttime gatherings.

### Outcomes

We compared the simulated HIV epidemic prior to model modification and after adding *disco matanga* to the model as described above. We then compared the model that incorporated *disco matanga* in scenarios with and without biomedical, biobehavioral, and structural interventions. Each scenario was run 100 times, each time with unique sets of parameters obtained from model calibration. The 100 parameter sets were identical for each scenario except for the addition of that scenario’s interventions. Each scenario was compared to a counterfactual in which no interventions for *disco matanga* were included. We compared HIV infections and deaths for adults ages 15 + in each scenario compared to its counterfactual and reported a percentage difference in infections and deaths over the period 2025–2050. We generated 95% confidence intervals from the 100 simulations using the Python package scipy.stats.bootstrap.

### Sensitivity analysis

We assessed the impact of variation of parameters listed in Table 1 on HIV attributable deaths in the period 2000–2050. We considered the variation of the condom usage probability, the duration of a *disco matanga* party, and the number of attendees at a *disco matanga* party in absence of the proposed interventions. We considered condom uptake at the parties of 0% (main analysis), 35%, and 70%. The duration of a *disco matanga* party in our main analysis was 14 days; to assess the impact this parameter has on the HIV transmission trend, we considered scenarios where the *disco matanga* party duration was 7 days for a shorter duration and 21 days for a longer duration. The *disco matanga* party size in our main analysis was 30 attendees; we considered the scenarios where the attendees were 15 for a smaller party size and 60 attendees for a larger party size. Results of sensitivity analysis were reported as a percent change in the primary outcome (HIV deaths) and visualized using a tornado plot.

## Results

We incorporated sexual interactions at *disco matanga* events into a previously developed network-based HIV transmission model and compared *disco matanga* scenarios of with or without the biomedical, biobehavioral, and structural interventions. We found that, since the start of the HIV pandemic, *disco matanga* has contributed an estimated 7.8% (95% CI: 5.5–9.3%) of HIV infections in Nyanza region. The contribution of *disco matanga* to new HIV infections peaked in 2004 at 9.9% (95% CI: 6.4–12.0%) of all new infections, corresponding to a peak in all-cause mortality rates—the triggering event for a *disco matanga*—which was in turn driven by the HIV/AIDS pandemic.

**Fig. 2:**
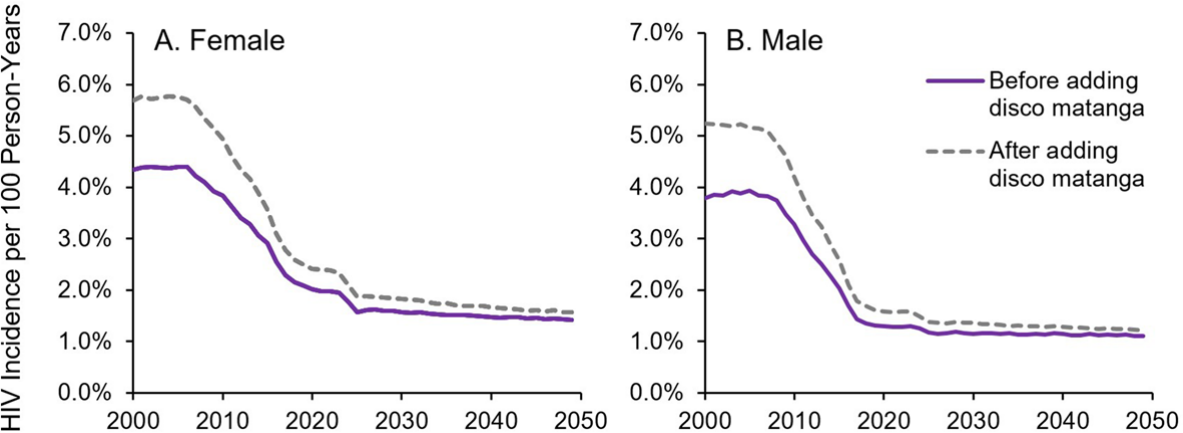
HIV incidence in **A** female and **B** male adults ages 15–49, before and after incorporating disco matanga into EMOD-HIV. Incidence is shown for all adults in the population, ages 15 + , regardless of current disco matanga attendance

Trends in HIV over time and by sex were similar with and without the incorporation of *disco matanga* (Fig. 2). Overall adult HIV incidence peaked over the period 2000–2006—the height of the HIV/AIDS pandemic in Kenya—before declining over the period 2006–2018 with the scale-up of HIV treatment and VMMC. After this period, HIV incidence was projected to decline more gradually through 2050, reflecting our assumption of continuation of present-day rates of engagement in HIV testing, care, and prevention.

**Fig. 3:**
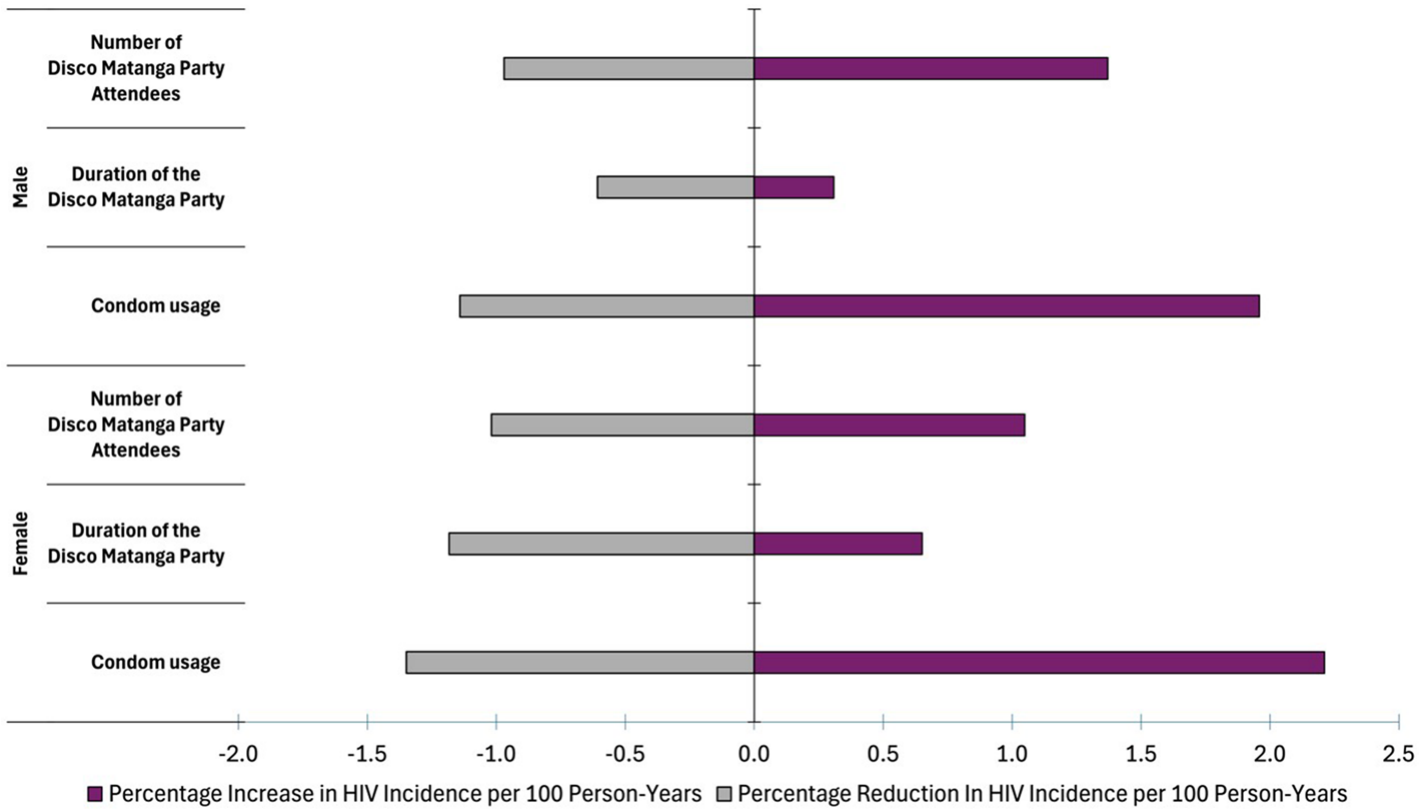
Sensitivity of HIV infections in western Kenya (2000–2050) to key assumptions regarding *disco matanga*: condom usage probability, duration of *disco matanga*, and size of the *disco matanga* party. Gray bars represent the difference in the outcome between the main analysis and the more conservative value chosen for sensitivity analysis, i.e., when decreasing *disco matanga* party size from 30 to 15, decreasing *disco matanga* party duration from 14 to 7 days, or increasing condom usage from 35 to 70%. Purple bars represent the difference in the outcome between the main analysis and the more extreme value chosen for sensitivity analysis, i.e., when increasing *disco matanga* party size from 30 to 60, increasing *disco matanga* party duration from 14 to 28 days, and decreasing condom usage from 35 to 0%. Results are stratified by male vs. female sex (top vs. bottom of graph)

The effect of incorporating *disco matanga* into the model was sensitive to uncertainties in key assumptions regarding *disco matanga*. For example, doubling the number of the *disco matanga* attendees from 30 to 60 per event led to an additional 1.1% increase in HIV infections among women and an additional 1.4% increase in HIV infections among men over the period 2000–2050. Halving the number of attendees from 30 to 15 led to a 1.0% decrease in HIV infections among both men and women. Sensitivities were similar in magnitude across the parameters explored (Fig. 3). The largest uncertainty was observed when reducing condom usage at *disco matanga* from 35 to 0%, leading to an additional 2.2% increase in HIV infections among women and 2.0% among men. HIV-related deaths were similarly sensitive to assumptions regarding *disco matanga*, but differed in that number of attendees per *disco matanga* event contributed more to uncertainty than did condom usage (Supplementary Fig. S1).

**Fig. 4:**
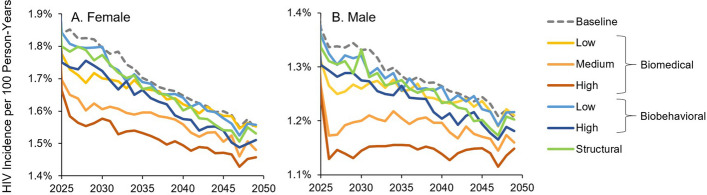
Effect of biomedical, biobehavioral, and structural interventions on HIV incidence in western Kenya. HIV incidence per 100 person-years in women (**A**) and men (**B**) ages 15 + is shown for a baseline intervention with disco *matanga* and no interventions (gray dashed line) and for biomedical, biobehavioral, and structural interventions designed to reduce HIV incidence

HIV prevention interventions targeted at *disco matanga* reduced HIV incidence among adults ages 15 + in the overall population to different extents (Fig. 4, solid lines). Low to moderate coverage of biomedical interventions for attendees (yellow and orange lines) produced incidence declines on par with the simulated biobehavioral and structural interventions (blue and green lines).

The hypothetical maximum impact of HIV prevention, i.e., providing highly effective biomedical prevention to 100% of attendees, produced an absolute reduction in HIV incidence of 0.05–0.1 per 100 person-years, compared to the no-intervention baseline.

**Fig. 5:**
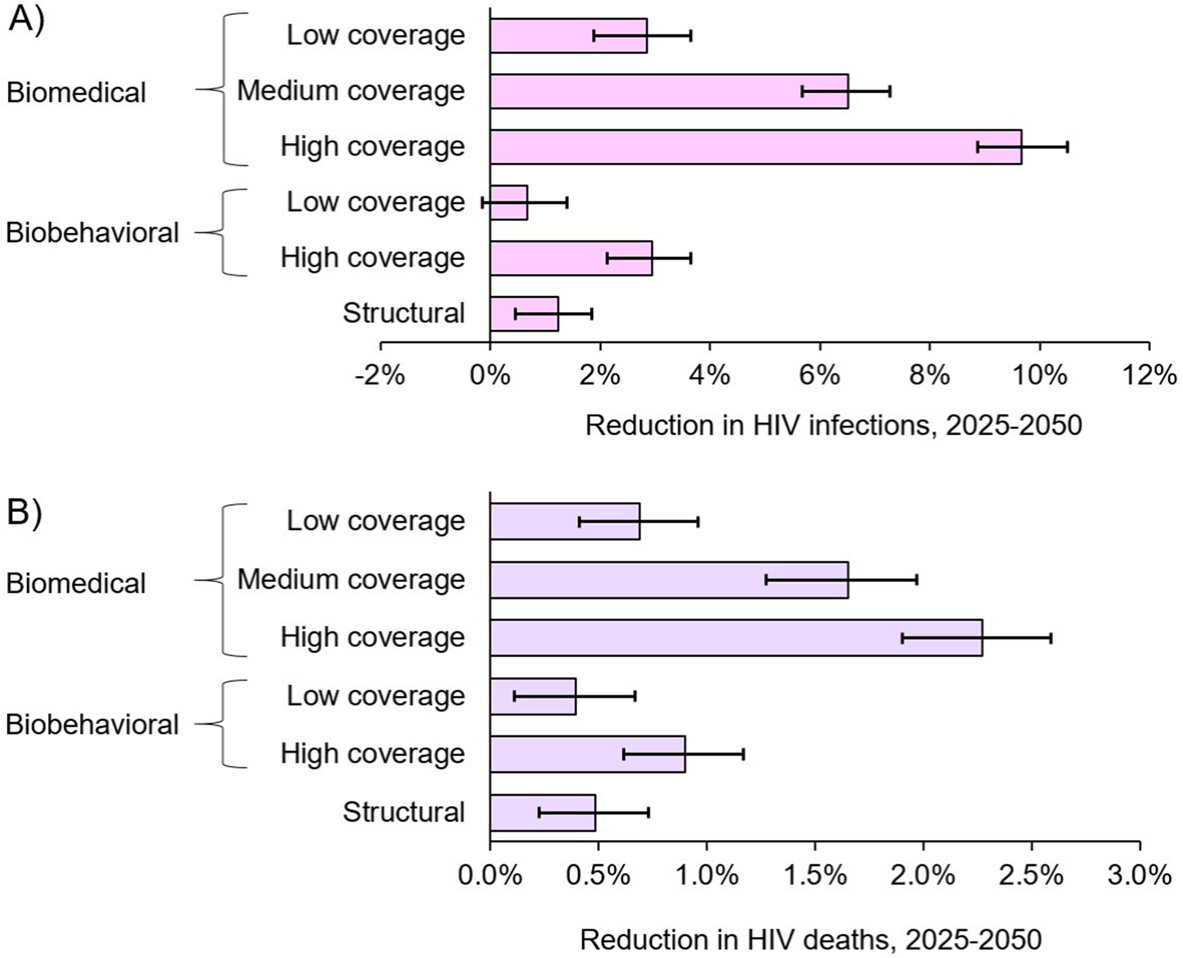
Impact of biomedical, biobehavioral, and structural interventions on HIV infections and deaths in western Kenya over the period 2025–2050*.* Impacts shown are a percent reduction in **A** cumulative HIV infections and **B** cumulative HIV-caused deaths over the period 2025–2050 relative to a scenario with no interventions

Relative reductions in 2025–2050 HIV infections and deaths with interventions for *disco matanga*, compared to no interventions, were substantial (Fig. 5) despite *disco matanga* interventions covering only a small fraction of all sexual activity.

Biomedical prevention at *disco matanga* could avert up to 9.67% (95% CI: 8.9–10.5%) of adult HIV infections and up to 2.27% (95% CI: 1.9–2.6%) of deaths if provided to all participants. Biobehavioral interventions could avert up to 2.9% (95% CI: 2.1–3.6%) of infections and 0.9% (95% CI: 0.6–1.2%) of deaths if 90% of participants practice safe sexual behaviors. Structural interventions could avert up to 1.2% (95% CI: 0.5–1.8%) of infections and 0.5% (95% CI: 0.2–0.7%) of deaths if they reduce sexual participation by one-third, e.g., by empowering young women to avoid a majority of unwanted sexual encounters.

In sensitivity analysis, impacts were sensitive to assumptions regarding intervention uptake (Fig. 5). When biomedical intervention uptake was reduced to 70%, the proportion of infections averted over 2025–2050 fell to 6.5% (95% CI: 5.7–7.3%) and the proportion of deaths averted fell to 1.7% (95% CI: 1.3–2.0%). When biomedical intervention uptake was further reduced to 30%, the proportion of HIV infections averted fell to 2.8% (95% CI: 1.9–3.7%) and the proportion of deaths averted fell to 0.7% (95% CI: 0.4–1.0%). Similarly, when biobehavioral intervention uptake was reduced to 10%, infections averted fell to 0.67% (0.81–0.73%) and the proportion of deaths averted fell to 0.67% (0.81–0.73%).

## Discussion

This mathematical modeling study of western Kenya is the first attempt, to our knowledge, to quantify the role of *disco matanga* in the HIV/AIDS pandemic and estimate the potential impact of biomedical, biobehavioral, and structural interventions. We found that a non-trivial proportion of all HIV infections (approximately 2–7%) could be averted by providing effective biomedical prevention for *disco matanga* attendees, such as oral or injectable chemoprophylaxis. A smaller but still substantial proportion (approximately 0.5–4%) could be averted through biobehavioral and structural interventions, which may include increases in condom use, reductions in drug and alcohol use, and/or avoidance of unwanted sexual encounters. Although addressing HIV risk at *disco matanga* will not be sufficient to end the HIV/AIDS pandemic, it may be an efficient way to target individuals and time periods of elevated HIV risk, potentially adding an important component to broader, more comprehensive HIV prevention programs.

To date, authorities in areas where *disco matanga*is practiced have attempted to reduce sexual risk through regulations such as banning nighttime activities and participation by children [8], and regulating locally produced alcoholic beverages such as beer (*busaa*) and spirits (*changaa*) [50]. However, these regulations vary in space and time, e.g., Kenya’s ban on *changaa*were reversed in 2010 due to instances of poisoning due to toxic compounds formed during unregulated production [51]. Enforcement of regulations on these activities has been variable and challenging, as *disco matanga* events typically occur in remote rural areas and communities that consider this practice culturally important are disincentivized to report to authorities. HIV biomedical, biobehavioral, and structural interventions could possibly provide an alternative pathway to reduce HIV risk associated with *disco matanga*in manners that benefit communities without the need for punitive measures. Recent studies have shown promising results including aversion of approximately 54% of HIV infections and 17% of HIV-related deaths in western Kenya as a result of 29% uptake of PrEP [30, 52]. For this reason, we assessed scenarios where there was increase PrEP uptake among the *disco matanga* attendees.

Our modeling study suggests that *disco matanga* events carry substantial HIV risk in areas with widely disseminated HIV epidemics and may require a multisectoral approach to reduce transmission. Deeply rooted gender norms, sexual risk at *disco matanga* is not only a matter of public health, but also one of gender equity [5–7]. Given reports of coerced and transactional sex at disco matanga, there is need to design and adopt women-centered empowerment programs, and ultimately, to break poverty cycles that create vulnerabilities leading to unwanted sexual exposures. Sensitization and education involving event organizers, community leaders, attendees, youth, and parents/caregivers could facilitate problem-solving around sexual coercion and transactional sex, while promoting the use of safe sexual practices and HIV prevention measures. *Disco matanga*events may represent unique, culturally tailored opportunities for HIV prevention and warrant empirical studies in the context of broader HIV prevention programs. Though focused on Kenya, the HIV biomedical, biobehavioral, and structural interventions investigated in this study could have applications to funeral rituals in other contexts, particularly settings where funerals practices could increase the risk of HIV transmission and acquisition. For example, in some South African communities, a post-funeral “after tears” party involves overnight alcohol consumption among youth, creating a high-risk environment for HIV transmission [53].

This study has several important limitations. First, any modeling exercise is only as strong as its assumptions. We developed model assumptions based on scant literature, supplemented by wide sensitivity analysis across uncertain parameters and contextual expertise of our authorship team, some of whom have direct experience with the cultural practices described. Our estimates may require revisiting as data accrue regarding demographics and risk around *disco matanga*, but at present they stand as the only estimates, to our knowledge, of the role of *disco matanga* in the HIV/AIDS pandemic. Second, like any modeling endeavor, we made simplifications and limitations to the scope of the analysis. We did not conduct sensitivity analyses regarding every aspect of the interventions modeled (uptake, efficacy, timeliness) but performed one illustrative sensitivity analysis of coverage to demonstrate how estimated impacts are sensitive to assumptions. Importantly, we assessed only direct HIV-related outcomes of *disco matanga* (HIV infections, HIV-related deaths, impact on prevalence) and did not include other outcomes such as unplanned pregnancies, sexually transmitted infections, and physical and emotional trauma. Though out of scope for the current modeling project, these outcomes may be impacted by *disco matanga* and warrant further investigation. Third, for future projections, we assumed continuation of current rates of HIV diagnosis, care engagement, VMMC, HIV prophylaxis use, and use of condoms in the general population. We did not incorporate more optimistic assumptions related to anticipated advances in HIV care and prevention (e.g., scale-up of long-acting injectable treatment and prophylaxis), nor did we include potential risks to maintaining high levels of HIV service coverage given declines in international HIV/AIDS financing. We made this “middle ground” assumption in order to focus our analyses on the specific theme of *disco matanga*, but we acknowledge that the many potential future scenarios for the broader set of HIV services provided in Kenya are a rich area for future study. Fourth, we assumed that biomedical interventions would have no effect on sexual behavior. While some studies have observed that participation in HIV pre-exposure prophylaxis programs reduced sexual risk behavior among young women in Kenya [54–56], others have observed behavioral disinhibition (i.e., increased unsafe sexual practices) in various contexts [18, 19]. Behavior change after accessing biomedical interventions has not, to our knowledge, been studied in the *disco matanga* context, and we opted for a neutral, “middle ground” assumption of no behavior change. However, it is noteworthy that our study also includes biobehavioral and structural interventions aimed at reducing unsafe sexual practices, which could be used to offset disinhibition effects if observed. Fifth, we did not measure the resource or feasibility constraints associated with the modeled interventions. Kenya is a resource-constrained setting with limited budget for healthcare and economic development. Some interventions, such as long-acting HIV prophylaxis, face global supply constraints. Additionally, there is a lack of evidence to inform demand and acceptability of biomedical, biobehavioral, and structural interventions for *disco matanga* attendees. Stigma, peer pressure, convenience, concerns about pain or side effects (for biomedical interventions), and other factors may limit the extent to which interventions can be adopted. Finally and importantly, we modeled interventions that, to our knowledge, have never been empirically tested. The present study could be used as motivation and justification for future empirical research, but numerical results from this study should be used with extreme caution until empirical data become available.

## Conclusions

*Disco matanga* presents a complex intersection of traditional practices, socio-cultural norms, and public health challenges. This study found that *disco matanga* has substantially contributed to the HIV/AIDS pandemic in western Kenya, and further, that a substantial proportion of future HIV infections in the region could be averted with effective biomedical, biobehavioral, or structural interventions. However, results were sensitive to the size and behavior within *disco matanga*, and to intervention uptake, and results were not informed by empirical testing. Research is needed to understand the impact, feasibility, acceptability, and cost-effectiveness of HIV interventions tailored to local cultural practices.

## Supplementary Information

Additional file 1: Supplementary Fig. S1 Sensitivity of HIV-caused deaths in western Kenya (2000–2050) to key assumptions regarding disco matanga: condom usage probability, duration of disco matanga, and size of the disco matanga party. Gray bars represent the difference in the outcome between the main analysis and the more conservative value chosen for sensitivity analysis, i.e., when decreasing disco matanga party size from 30 to 15, decreasing disco matanga party duration from 14 to 7 days, or increasing condom usage from 35 to 70%. Red bars represent the difference in the outcome between the main analysis and the more extreme value chosen for sensitivity analysis, i.e., when increasing disco matanga party size from 30 to 60, increasing disco matanga party duration from 14 to 28 days, and decreasing condom usage from 35 to 0%. Results are stratified by male vs. female sex (top vs. bottom of graph).

## Data Availability

Mathematical model source code that was used to generate the findings of this study is available at https://github.com/InstituteforDiseaseModeling/EMOD.

## Abbreviations

AIDS: Acquired immunodeficiency syndrome
EMOD: Epidemiological MODeling software
HIV: Human immunodeficiency virus
VMMC: Voluntary male medical circumcision
