# Supplementary material for: Estimated contributions and future mitigation strategies for HIV risk around funeral practices in western Kenya: a mathematical modeling study
Source: BMC Med. 2025 Feb 12;23:85. doi: 10.1186/s12916-025-03907-4 (PMC11823116; doi:10.1186/s12916-025-03907-4)
Supplement: Supplementary file 1 — Additional file 1: Supplementary Fig. S1 Sensitivity of HIV-caused deaths in western Kenya (2000–2050) to key assumptions regarding disco matanga: condom usage probability, duration of disco matanga, and size of the disco matanga party. Gray bars represent the difference in the outcome between the main analysis and the more conservative value chosen for sensitivity analysis, i.e., when decreasing disco matanga party size from 30 to 15, decreasing disco matanga party duration from 14 to 7 days, or increasing condom usage from 35 to 70%. Red bars represent the difference in the outcome between the main analysis and the more extreme value chosen for sensitivity analysis, i.e., when increasing disco matanga party size from 30 to 60, increasing disco matanga party duration from 14 to 28 days, and decreasing condom usage from 35 to 0%. Results are stratified by male vs. female sex (top vs. bottom of graph). [file 12916_2025_3907_MOESM1_ESM.docx]

**Supplementary Figure for:**

**Estimated contributions and future mitigation strategies for HIV risk around funeral practices in western Kenya: A mathematical modeling study**

Samuel M. Mwalili^1,2^, Duncan K. Gathungu^1,2^, Josiline Chemutai^1^, Evalyne Musyoka^1^, Daniel Bridenbecker^3^, Clark Kirkman IV^3^, David Kaftan^4^, Hae-Young Kim^4^, Ingrida Platais^4^, Anna Bershteyn^4§^

^1^Strathmore University, Ole Sangale Road, P.O. Box 59857-00200, Nairobi, Kenya.
^2^Jomo Kenyatta University of Agriculture and Technology, P.O. Box 62000-00200, Nairobi Kenya. ^3^Institute for Disease Modeling at the Bill & Melinda Gates Foundation, 500 5^th^ Avenue North, Seattle, Washington, USA.
^4^New York University Grossman School of Medicine, 227 East 30^th^ St., New York, New York, USA.


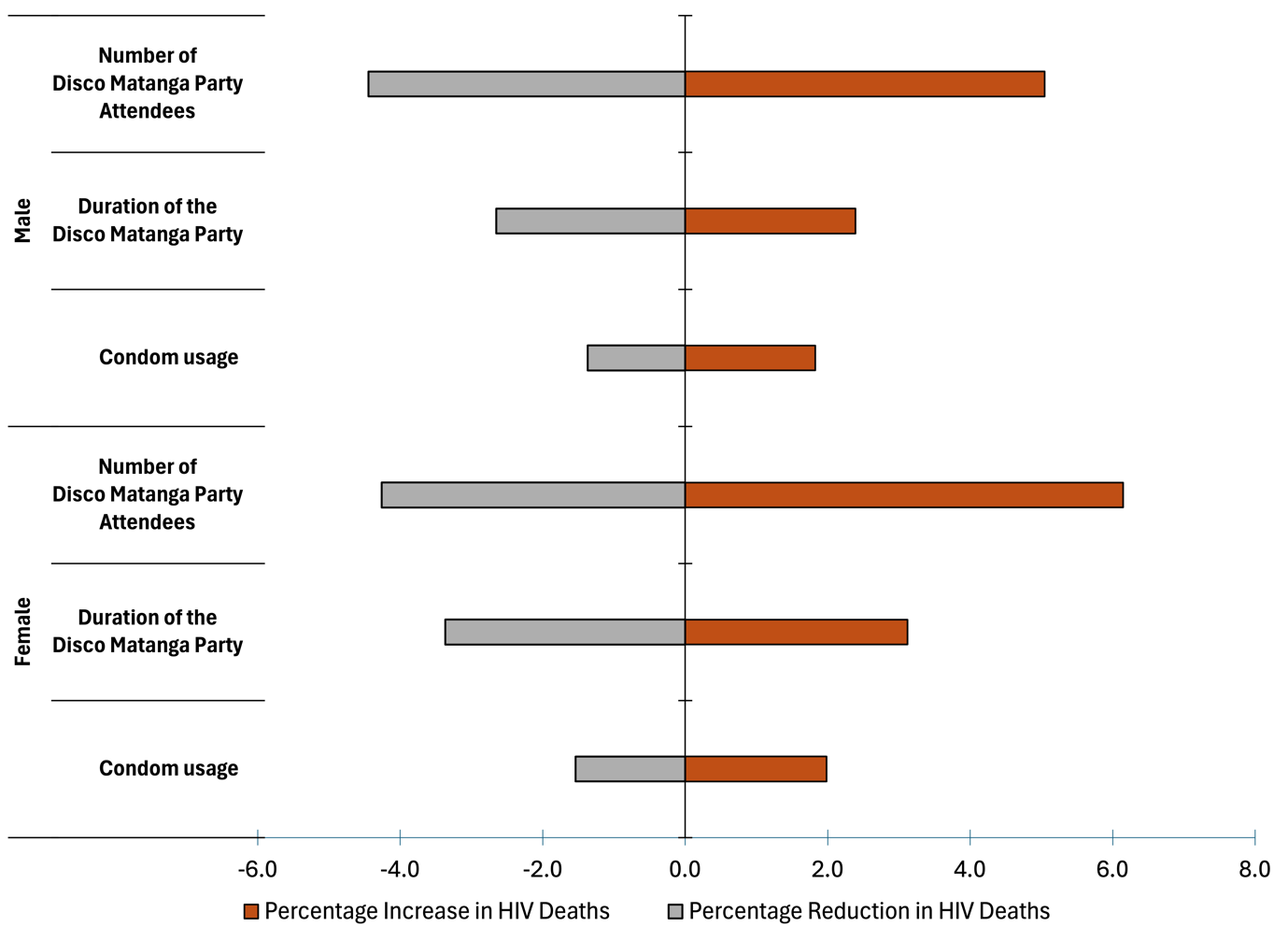


**Supplementary Figure S1: Sensitivity of HIV-caused deaths in western Kenya (2000-2050) to key assumptions regarding disco matanga: condom usage probability, duration of disco matanga, and size of the disco matanga party.** Grey bars represent the difference in the outcome between the main analysis and the more conservative value chosen for sensitivity analysis, i.e., when decreasing disco matanga party size from 30 to 15, decreasing disco matanga party duration from 14 to 7 days, or increasing condom usage from 35% to 70%. Red bars represent the difference in the outcome between the main analysis and the more extreme value chosen for sensitivity analysis, i.e, when increasing disco matanga party size from 30 to 60, increasing disco matanga party duration from 14 to 28 days, and decreasing condom usage from 35% to 0%. Results are stratified by male vs. female sex (top vs. bottom of graph).
